# Pharmacist-led management of chronic pain in primary care: costs and benefits in a pilot randomised controlled trial

**DOI:** 10.1136/bmjopen-2014-006874

**Published:** 2015-04-01

**Authors:** Aileen R Neilson, Hanne Bruhn, Christine M Bond, Alison M Elliott, Blair H Smith, Philip C Hannaford, Richard Holland, Amanda J Lee, Margaret Watson, David Wright, Paul McNamee

**Affiliations:** 1 Health Economics Research Unit, University of Aberdeen, Aberdeen, UK; 2 Centre of Academic Primary Care, University of Aberdeen, Aberdeen, UK; 3 Division of Population Sciences, University of Dundee, Dundee, UK; 4 Norwich Medical School, University of East Anglia, Norwich, UK; 5 Medical Statistics Team, University of Aberdeen, Aberdeen, UK; 6 School of Pharmacy, University of East Anglia, Norwich, UK

**Keywords:** HEALTH ECONOMICS, PAIN MANAGEMENT, PRIMARY CARE

## Abstract

**Objectives:**

To explore differences in mean costs (from a UK National Health Service perspective) and effects of pharmacist-led management of chronic pain in primary care evaluated in a pilot randomised controlled trial (RCT), and to estimate optimal sample size for a definitive RCT.

**Design:**

Regression analysis of costs and effects, using intention-to-treat and expected value of sample information analysis (EVSI).

**Setting:**

Six general practices: Grampian (3); East Anglia (3).

**Participants:**

125 patients with complete resource use and short form-six-dimension questionnaire (SF-6D) data at baseline, 3 months and 6 months.

**Interventions:**

Patients were randomised to either pharmacist medication review with face-to-face pharmacist prescribing or pharmacist medication review with feedback to general practitioner or treatment as usual (TAU).

**Main outcome measures:**

Differences in mean total costs and effects measured as quality-adjusted life years (QALYs) at 6 months and EVSI for sample size calculation.

**Results:**

Unadjusted total mean costs per patient were £452 for prescribing (SD: £466), £570 for review (SD: £527) and £668 for TAU (SD: £1333). After controlling for baseline costs, the adjusted mean cost differences per patient relative to TAU were £77 for prescribing (95% CI −82 to 237) and £54 for review (95% CI −103 to 212). Unadjusted mean QALYs were 0.3213 for prescribing (SD: 0.0659), 0.3161 for review (SD: 0.0684) and 0.3079 for TAU (SD: 0.0606). Relative to TAU, the adjusted mean differences were 0.0069 for prescribing (95% CI −0.0091 to 0.0229) and 0.0097 for review (95% CI −0.0054 to 0.0248). The EVSI suggested the optimal future trial size was between 460 and 690, and between 540 and 780 patients per arm using a threshold of £30 000 and £20 000 per QALY gained, respectively.

**Conclusions:**

Compared with TAU, pharmacist-led interventions for chronic pain appear more costly and provide similar QALYs. However, these estimates are imprecise due to the small size of the pilot trial. The EVSI indicates that a larger trial is necessary to obtain more precise estimates of differences in mean effects and costs between treatment groups.

**Trial registration number:**

ISRCTN06131530.

**Strengths and limitations of this study**

- This is the first study to assess the differences in costs and effects (in terms of quality-adjusted life years (QALYs)) associated with pharmacist prescribing and/or pharmacist review compared with treatment as usual (TAU) in the management of chronic pain in primary care.
- The results are imprecise as they were based on a pilot randomised controlled trial and a larger trial based on these results is now needed.
- Using a value of information approach to determine optimal sample, findings suggest a future trial requires between 460 and 690, and between 540 and 780 patients per arm using a threshold of £30 000 and £20 000 per QALY gained, respectively, in order to obtain more precise estimates of differences in mean QALYs and mean costs between treatment groups.

## Introduction

Chronic pain is recognised as a common and long-term condition having a significant impact on the quality of life of individuals and their families, along with a considerable financial cost to society. Its pathophysiology and psychosocial impact are largely independent of its biological aetiology and therefore, comprehensive approaches to identifying, designing and targeting relevant interventions have been advocated, whereby chronic pain is dealt with as a whole clinical entity, regardless of its underlying cause.^1^ Chronic pain is characterised by pain which persists despite adequate time for healing. Although there is no clear definition, it is often defined as pain that has been present for more than 3 months.^2^ Approximately 20% of the European adult population have chronic pain^3^ and the economic burden is considerable with national healthcare and socioeconomic costs running into billions annually, representing between 3% and 10% of European gross domestic product (GDP)^4^ and from $560 to $635 billion annually in the USA.^5^ In the UK, an estimated 11%^6^ of adults suffer from severe chronic pain, representing around 4.5 million people. The average annual incidence risk estimated from health surveys of developing chronic pain is 8.3% and for those already with chronic pain (ie, prevalent cases), the average annual recovery rate is 5.4%.^7^ The total cost of back pain alone has been estimated to be £12.3 billion annually in the UK or 1.5% of GDP.^8^

The majority of patients with chronic pain are managed outside the specialist setting, mainly in primary care; however**,** patients will make a variety of choices for self-management of their pain.^3^ Pharmacological and non-pharmacological management options exist with provision of the former involving mostly primary care or purchase of over-the-counter medicines from the community pharmacy or supermarket; the latter options include cognitive behavioural therapy, physiotherapy or alternative therapies and are often provided privately. The recent Scottish Intercollegiate Guidelines Network (SIGN) guideline on chronic pain^1^ reviewed the evidence for assessment and management of chronic non-malignant pain in the adult population in the non-specialist setting and reported on the current lack of good quality evidence to guide management. The guideline indicated a range of recommendations relating to pharmacological therapies including, for example, the provision of at least an annual assessment of patients on pharmacotherapy for chronic pain.

We recently reported findings from the ‘Pharmacist-led management of chronic pain in primary care’ (PIPPC) pilot trial in which pharmacist medication review with face-to-face pharmacist prescribing was compared with pharmacist medication review with feedback to the general practitioner (GP) or usual GP-led care.^9^ This was the first pilot randomised controlled trial (RCT) to compare key clinical outcomes (rather than process end points) between pharmacist-led interventions for adults with chronic pain with the usual GP care. Although it was a pilot trial to inform the design for a subsequent definitive trial, the findings suggested that compared to usual care, participants’ pain levels, as measured by the chronic pain grade (CPG), improved in both pharmacist-led intervention groups, particularly in the group managed by pharmacist prescribing. The pilot trial also collected data on resource use and health-related quality of life. This provided an opportunity to assess the impact of pharmacist-led interventions on health economic outcomes and to offer some insight into the potential value for money of the study interventions. The objective of this paper, therefore, is to calculate the differences in mean costs and effects of the interventions, and the precision of those estimates so as to inform sample size and other design considerations for a future definitive trial.

## Methods

### Overview of the PIPPC trial

The PIPPC study^9^ was a UK-based 6-month open exploratory pilot RCT primarily designed to investigate the effectiveness of either pharmacist medication review and face-to-face pharmacist prescribing, or pharmacist medication review and feedback to GP, relative to treatment as usual (usual GP-led care). The study was performed in six general practices with an attached independent pharmacist prescriber (3 in Grampian and 3 in East Anglia). Full details have been reported previously.^9^ Briefly, a total of 196 patients were recruited into the trial between March and June 2010. Included patients were: over 18 years of age; living in their own home; and receiving regular prescribed medication for pain (defined as receiving within the previous 120 days either two or more acute prescriptions, and/or one repeat prescription for an analgesic and /or a non-steroidal anti-inflammatory drug (NSAID)). Patients were randomised in a 1:1:1 ratio to receive either pharmacist medication review with pharmacist prescribing (n=70) or pharmacist review only (n=63) or TAU (n=63). Patients and their healthcare providers were unblinded due to the nature of the intervention. Trial outcomes assessed by self-report in postal questionnaires at baseline, and at 3 and 6 months’ follow-up included: the short form mental (SF-12 MCS) and physical (SF-12 PCS) component summary scores, the generic preference-based health state utilities measure short form-six-dimension questionnaire (SF-6D), the CPG, and the Hospital Anxiety and Depression Scale (HADS). Preference weights (utilities) for the SF-6D health status measure were derived using the recommended tariffs derived from a UK general population survey.^10^ Data on health service resource use were also collected at three time points: baseline (to measure service use 3 months prerandomisation), 3 months (to measure resource use 3 months postrandomisation) and 6 months (to measure resource use 3–6 months postrandomisation), as described below.^9^

### Resource use and unit costs

The economic analysis included the direct costs associated with the intervention (pharmacist training, pharmacist and GP time involved in delivering the intervention along with related follow-up), pain-related hospitalisation (number of hospital inpatient days, day cases and outpatient visits), primary care visits for chronic pain (GP, nurse, healthcare assistant appointments), primary care telephone contacts for chronic pain, prescribed and non-prescribed OTC pain-related medications.

Frequency of the above primary and secondary care visits, and names and quantity of medicine prescribed were measured and valued using information from patients’ medical records and by patient self-reported questionnaire. Standard unit costs from published UK sources were attached to these items of resource use. Resource use quantities and the assigned unit costs used in this analysis are summarised in table 1. For study medications, unit costs were obtained from the British National Formulary.^11^ Hospital inpatient and outpatient unit costs were based on average costs from the Scottish Health Service Costs Book.^12^ Intervention-related pharmacist and GP time were based on costs from the Personal Social Services Research Unit (PSSRU).^13^ Intervention-related costs (pharmacist training, etc.) was based on information obtained from follow-up semistructured interviews with pharmacists and GPs in participating practices.

**Table 1:** Unit costs applied to NHS resource use in the PIPPC study

Resource use item	Value (£)	Unit	Source/comments
Intervention costs			
Pharmacist training	Prescribing: £10.85 Review: £10.67	Per patient	PSSRU 2009/2010^13^ 2 days (ie16 h) @ £40/h (includes travel)=£40×16=£640, this cost being apportioned over the number of patients in each intervention group
Intervention delivery	Prescribing: £66.7 Review: £44	Per patient	PSSRU 2009/2010^13^ £40/h (includes travel) Prescribing: mean duration 100 min; Review: mean duration 66 min
Pharmacist follow-up appointments	Prescribing: £6.18 Review: £0	Per patient	PSSRU 2009/2010^13^ £40/h Prescribing: 45/53 (84.9%) patients received follow-up appointments: 0 (15.1%); 1 (62.3%) mean duration 11 min; 2 (15.1%) mean duration 10 min, 3 (7.5%) mean duration 12 min. Most were conducted by phone. Weighted average cost=£6.18 Review: no follow-up appointments
GP review	£45.9	Per patient	PSSRU 2009/2010^13^ £106/h of GMS activity. Mean duration 26 min
Primary care costs			
GP-home	£108	Per consultation	PSSRU 2009/2010^13^
GP-surgery	£32	Per consultation	PSSRU 2009/2010^13^
GP-telephone	£20	Per consultation	PSSRU 2009/2010^13^
Nurse-home	£20	Per consultation	PSSRU 2009/2010^13^
Nurse-surgery	£12	Per consultation	PSSRU 2009/2010^13^
Nurse-telephone	£7.50	Per consultation	PSSRU 2009/2010^13^ (based on an assumed multiplier for telephone consultations for nurse and healthcare assistant of 0.625, that is, 20/32
Other-home	£9	Per consultation	PSSRU 2009/2010^13^ (clinical support worker nursing—community)
Healthcare assistant-surgery consultation	£7.50	Per consultation	Based on an assumed multiplier for telephone consultations for nurse and healthcare assistant of 0.625, that is, 20/32
Hospital costs			
Inpatient stay	Orthopaedics=£873 General medicine=£395	Per bed day	ISD Scotland, Scottish Health Service Costs**,** NHS Grampian, year ended 31 March 2010^12^ R040 (&LS)—Specialty Group Costs—Inpatients
Day case	Pain relief=£541	(Net) per case	ISD Scotland, Scottish Health Service Costs**,** NHS Grampian, year ended 31 March 2010^12^ R042—Specialty Costs—Day Cases
Outpatient clinic—consultant led	Pain relief=£122	Per attendance	ISD Scotland, Scottish Health Service Costs**,** NHS Grampian, year ended 31 March 2010^12^ R044—Specialty Costs—Consultant Outpatient Clinics
Outpatient clinic—other (eg, physiotherapist)	Pain relief=£37	Per attendance	ISD Scotland, Scottish Health Service Costs**,** NHS Grampian, year ended 31 March 2010^12^ R046—Specialty Costs—AHP Outpatient Clinics
Medications	Varied by medication	Dose, duration, pack size, number supplied etc	BNF 61, March 2011^11^

PIPPC study, Pharmacist-led management of chronic pain in primary care; PSSRU, Personal Social Services Research Unit; NHS National Health Service.

Other costs borne by patients (eg, OTC medicines, travel) or their carers, and productivity losses to society were considered outside the scope of the National Health Service (NHS) perspective of the present analysis. All costs were in UK pounds sterling (£), undiscounted (as the duration of the trial was less than 1 year). The price year used for all costs was 2009/2011.

### Effects

The effects of the pharmacist-led interventions were estimated as gain in quality-adjusted life years (QALYs). Based on the SF-6D data collected at baseline, 3**-**month and 6-month follow-up, the number of QALYs over 6 months follow-up associated with each study arm were approximated by applying the area under the curve (AUC) method (implemented by summing the areas of the geometrical shapes obtained by linearly interpolating between utility scores over the study period).^10^ QALYs were undiscounted due to the short duration of the trial.

### Methods used to estimate differences in mean costs and effects

Similar to the main analysis of clinical outcomes,^9^ the economic analysis was conducted on an intention-to-treat basis for participants with complete SF-6D data at baseline, 3-month and 6-month follow-up. While resource use information was available for 178 patients, only 125 patients had complete SF-6D data for all three study assessment time-points. These 125 patients formed the sample for the main analysis. Using the data described above, estimates of the total mean costs and total mean QALYs per group were calculated. Regression analyses calculated the differences (and associated 95% CIs) in mean total costs and differences in mean total QALYs per patient, while controlling for differences in total costs at baseline, baseline SF-6D^14^ and other baseline patient characteristics (age, sex, marital status, work status, education, income, baseline CPG—intensity).

### Sensitivity analyses

We performed a number of sensitivity analyses to explore areas of uncertainty in the main analysis. First, as noted above approximately one-third of patients were excluded due to missing some SF-6D data at follow-up (no missing cost data was apparent and so imputation was not necessary). To assess whether this introduced bias, we performed a sensitivity analysis with imputed values for missing SF-6D data and re-ran the regression analysis using data from all 178 patients. The missing SF-6D data were imputed by multiple imputation via chained equations with the STATA 13 ‘mi impute’ command programme to account for patients with missing data at either 3 or 6 months assuming that data were missing at random (and creating five imputed data sets).^15^ ^16^ Second, to investigate the impact on differential costs from patients who experienced very high costs from the analysis, we conducted an analysis excluding hospital inpatient costs, on the basis that some of these costs may not be directly related to chronic pain. Finally, we explored whether the main results were affected by controlling only for baseline differences between groups in total costs and SF-6D (ie, excluding sociodemographic and economic factors, and other health status measures, ie, the CPG).

### EVSI analysis

The expected value of sample information (EVSI)^17^ ^18^ was calculated to assess whether conducting a larger RCT of pharmacist prescribing versus TAU, or pharmacist review versus TAU would be worthwhile. This approach determines the optimal sample size that maximises the difference between the expected total cost (Total Cost) of a future trial and the monetary value of the information that it provides (EVSI). The EVSI is the anticipated value of the health gain (or the expected net benefit of sample information (‘Expected Net Gain’, ENG)) over the period of t years, the chosen time horizon used for the analysis. This time horizon represents how long we expect the ‘new intervention(s)’ to become established as the ‘standard of care in the UK’ should it be shown to be cost effective. Total Cost is categorised into three main elements: a fixed component to represent the human (ie, labour/staff resource use) and other resources associated with the setting up and running of the trial; a variable component to reflect per patient accrual (ie, per additional patient recruited or increases in each participant taking part and who might potentially benefit from the intervention), follow-up and data collection costs; and an opportunity cost—health loss to participants randomised to the control group (assuming the intervention is effective). To generate EVSI estimates, the difference in effectiveness, costs and associated variances and covariances observed within the PIPPC trial were used. In addition, assumptions were made regarding trial fixed costs, variable costs and the number of future patients with chronic pain expected to benefit from the proposed pharmacist prescribing and review intervention. For our analysis, we made the following assumptions: time horizon=30 years; annual incidence=540 000 (this value was calculated based on the average annual incidence of chronic pain is 8.3% in adults aged 25 years and older,^7^ and the number of adult population is around 42 million (last UK census) that gives 3.5 million. This implies that approximately 1.2 million (1/3×3.5 million) people develop chronic pain that is ongoing/long-standing (ie lasting >3months). On the basis of the PIPPC trial,^9^ only a proportion of these people were receiving medication—we calculate this to be 1492 people from a total of 3281 or 45%. So, the assumed incidence used 540 000); a fixed trial cost=£1.4 million; and a variable trial cost=£220. We generated EVSI estimates using a willingness to pay per QALY gained of £20 000 and £30 000, respectively.

Additional sensitivity analyses checked whether the EVSI results were robust to variations in the main analysis assumptions, including the underlying incidence rate, the number of patients who would benefit and the recruitment cost per patient to a trial. Specifically, we halved and doubled the values used in the main analysis in each alternative case, with the exception of time horizon (10/20 years).

## Results

### Resource use and costs

Table 2 presents the number of patients using each type of resource (and percentages), and per patient mean quantities used, mean unadjusted cost per type of resource, unadjusted total mean costs and the adjusted cost differences by trial arm, from baseline to 6-month follow-up. As the table shows, there was some variation in the resources used and associated costs between patients in each study arm. For example, the results suggest that GP resource use is higher in the review group relative to either prescribing or TAU groups, and also that nurse practice consultations by phone is higher in the prescribing group relative to review and TAU.

**Table 2:** Mean NHS resources used and associated costs per patient from baseline to 6 months follow-up in the PIPPC study (complete case analysis n=125)

	Prescribing (n=39)			Review (n=44)			TAU (n=42)		
Resource use item	n (%)	Quantity	Cost, £	n (%)	Quantity	Cost, £	n (%)	Quantity	Cost, £
Intervention-related									
Pharmacist training (hours)	39 (100)	16*	10.9	44 (100)	16*	10.7	NA	–	–
Pharmacist activities related to intervention delivery (mins)	39 (100)	100†	66.7	44 (100)	66‡	44	NA	–	–
Pharmacist follow-up (mins)	39 (100)	9.3§	6.2	44 (100)	0	–	NA	–	–
GP review of pharmacist recommendations (mins)	NA	`	–	44 (100)	26	45.9	NA	–	–
Primary care									
GP consultations-surgery	25 (64)	2.6	84.5	32 (73)	3.5	113	30 (71)	3.2	102.4
GP consultations-phone	4 (10)	1.3	25	13 (30)	1.8	35.3	8 (19)	2.4	47.5
GP consultations-home	0 (0)	–	–	1 (2)	1.0	108	1 (2)	6.0	648
Practice nurse consultations-surgery	9 (23)	2.4	29.3	10 (23)	2.7	32.4	9 (21)	2.0	24
Practice nurse consultations-home	0 (0)	–	18	0 (0)	–	–	1 (2)	1.0	20
Practice nurse consultations-phone	13 (33)	1.6	12.11	3 (7)	1.7	12.5	3 (7)	1.0	7.5
HCA-home	1 (3)	2.0	18	2 (5)	2.0	18	3 (7)	1.3	12
HCA-surgery	9 (23)	4.6	34.2	13 (30)	3.7	27.7	11 (26)	1.8	13.64
Secondary care									
Hospital inpatient stay (days)	2 (5)	2.0	790	2 (5)	3.5	1621	1 (2)	2.0	790
Hospital inpatient day case	1 (3)	1.0	541	1 (2)	1.0	541	4 (10)	1.0	541
Hospital outpatient-consultant led	7 (18)	2.9	348.6	14 (32)	2.0	244	15 (36)	2.6	317.2
Hospital outpatient-other	6 (15)	1.8	67.8	7 (16)	2.7	100.4	4 (10)	3.3	120.3
Medications	39 (100)	Various¶	165.2	43 (98)	Various¶	181.4	42 (100)	Various¶	364.9
Unadjusted total costs, mean (SD)**,††	452.2 (466.0)†			569.7 (526.6)				668.2 (1333.4)	
Adjusted difference in total costs versus TAU, mean (95% CI)‡‡	77.5 (−81.7 to 236.7)§§			54.4 (−103.3 to 212.1)§§					

*Pharmacist training time apportioned across all patients in each study group.

†Pharmacist time (prescribing group) spent on tasks including: face-to-face consultations; record-based medication review, careplan preparation, meeting GP.

‡Pharmacist time (review group) spent on tasks including: record-based medication review, careplan preparation, and meeting GP.

§Based on PIPPC study data around 85% patients had (between 1 and 3) follow-up visits in the prescribing group with a weighted average duration across all patients of 9.3 min.

¶Owing to the large number of different types of medications used (total n=48) only summary cost details are presented here.

**Raw unadjusted mean total costs over the total patients per study group.

††For comparison the raw unadjusted (3 months period) prerandomisation total mean costs by study group were: prescribing=£364.8; review=£436.6; TAU=£624.7.

‡‡Estimates from regression analyses with adjustment for differences in baseline costs, baseline SF-6D health utility score and other patient characteristics (age, sex, marital status, work status, education, income, baseline CPG—intensity). The number of patients with data on all these baseline variables: prescribing (n=35); review (n=39), TAU (n=34).

§§Cost differences are calculated as the difference in cost between the intervention group (prescribing or review) and the TAU group.

CPG, chronic pain grade; GP, general practitioner; HCA, healthcare assistant; NHS, National Health Service; PIPPC study, Pharmacist-led management of chronic pain in primary care; TAU, treatment as usual.

**Figure 1:**
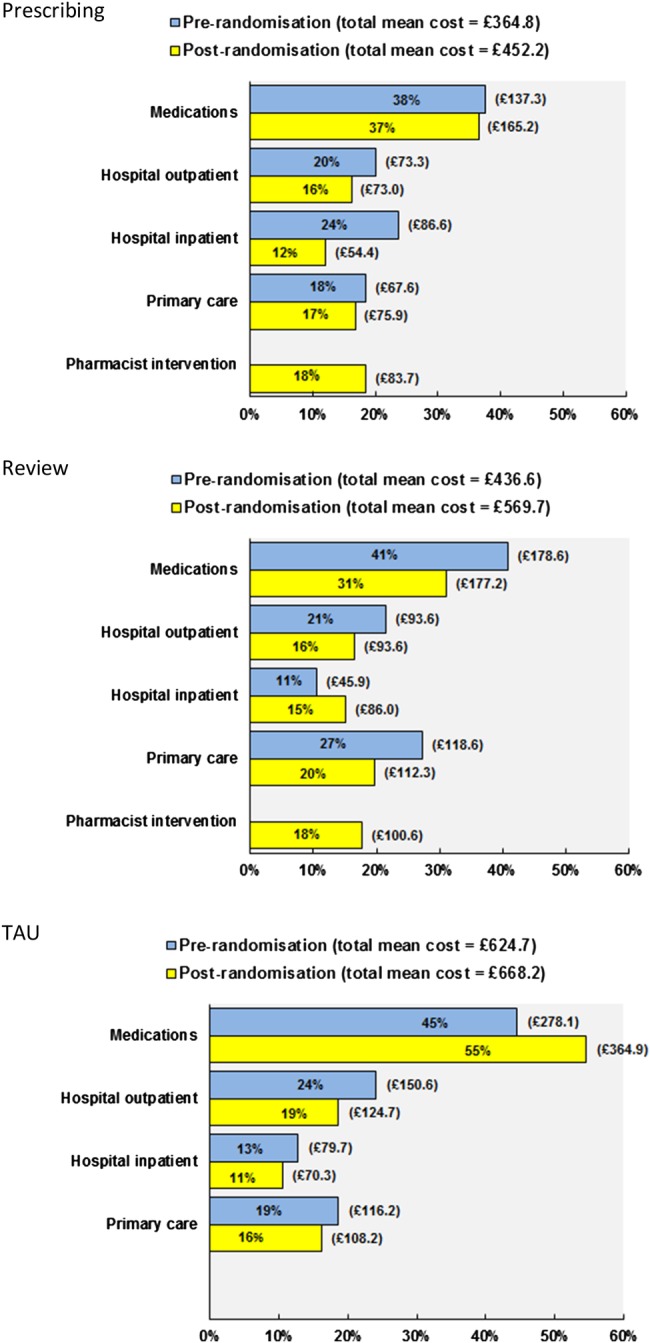
Proportion of unadjusted total mean costs per patient prerandomisation (ie baseline) and at 6 months follow-up, by each main cost component and study arm.

The bar charts in figure 1 show per patient the different cost components as a proportion of the total (unadjusted) costs in each study arm. At both baseline and follow-up, medications accounted for the largest percentage of the total cost in all study arms (prescribing 37%, review 31%, TAU 55%), outpatient hospitalisations for TAU (19%), intervention-related costs for pharmacist prescribing (18%) and primary care costs, excluding pharmacist visit costs, for pharmacist review only (20%). Both pharmacist-led intervention arms were less costly than TAU based on the raw unadjusted mean total costs. The TAU group, however, was also the most costly treatment group observed prerandomisation and this was largely driven by medication costs; this suggests potential imbalances between study arms at baseline (figure 1). Following adjustment for differences in baseline costs and controlling for other baseline patient characteristics (age, sex, marital status, work status, education, income, baseline CPG—intensity and baseline SF-6D) yielded positive incremental mean costs differences for both pharmacist-led interventions relative to TAU: prescribing £77.5 (95% CI −£81.7 to £236.7) and review £54.4 (95% CI −£103.3 to £212.1). In other words, the review and prescribing groups relative to TAU were now more expensive rather than cheaper, relative to usual care. Adjusting for baseline costs were largely responsible for this resulting change which was statistically significant (with a regression coefficient p=0.0000). No other variables reached significance.

### Quality-adjusted life years

Table 3 shows the SF-6D health utility scores by study arm. The values were broadly similar across all arms at each time point, although pharmacist prescribing showed slightly higher SF-6D scores at all three time points.

**Table 3:** SF-6D health utility scores and QALYs over 6 months follow-up in the PIPPC study (complete case analysis n=125)

	Prescribing (n=39)	Review (n=44)	TAU (n=42)
SF-6D at baseline, mean (SD)	0.6349 (0.1336)	0.6173 (0.1431)	0.6077 (0.1140)
SF-6D at 3 months, mean (SD)	0.6428 (0.1396)	0.6411 (0.1469)	0.6226 (0.1405)
SF-6D at 6 months*,* mean (SD)	0.6500 (0.1462)	0.6291 (0.1471)	0.6105 (0.1336)
Unadjusted total QALYs, mean (SD)	0.3213 (0.0659)	0.3161 (0.0684)	0.3079 (0.0606)
Adjusted difference in total QALYs versus TAU, mean (95% CI)*	0.0069 (−0.0091 to 0.0229)	0.0097 (−0.0054 to 0.0248)	

The number of patients with data on all these baseline variables: prescribing (n=35); review (n=39), TAU (n=34).

*Estimates from regression analyses with adjustment for differences in baseline costs, baseline SF-6D health utility score and other patient characteristics (age, sex, marital status, work status, education, income, baseline CPG—intensity).

QALYs, quality-adjusted life years; SF-6D, short form six-dimension; TAU, treatment as usual.

Both pharmacist-led intervention arms generated slightly more QALYs than TAU based on the raw unadjusted mean total QALYs, but the magnitude of QALY gains relative to TAU were small (approximately 0.01 extra QALYs) in both cases.

After adjusting for baseline SF-6D score, baseline costs and controlling for other baseline patient characteristics (age, sex, marital status, work status, education, income, baseline CPG—intensity), QALY gains relative to TAU were largely unchanged for pharmacist prescribing 0.0069 (−0.0091 to 0.0229) and pharmacist review 0.0097 (−0.0054 to 0.0248).

### Sensitivity analyses

The sensitivity analysis with missing SF-6D data imputed (ie, 125 out of 178 complete; 53 incomplete, so imputed) by using multiple imputations (MI) produced similar values to the main analyses for estimated difference in mean total QALYs; however, differences in QALYs were slightly larger for pharmacist prescribing versus TAU 0.0065 (−0.0075 to 0.0205) than pharmacist review versus TAU 0.0047 (−0.0086 to 0.0181). Using the full resource use data set and rerunning the cost regression analysis showed the two pharmacist-led interventions still to be more costly than TAU, though the magnitude of the adjusted difference in cost was reduced in the prescribing group and increased in the review group relative to TAU, prescribing £21 (−124 to £167) and review £75 (−72 to £221). Excluding from the total costs the few patients in each arm having hospital inpatient care (hospital stay and day cases) reduced raw unadjusted mean total costs per patient by around £90, £130 and £40 in the pharmacist prescribing, pharmacist review and TAU arms, respectively. The adjusted differences in mean total costs (after controlling for baseline: costs, SF-6D, age, sex, marital status, work status, education, income and CPG—intensity) were increased for both pharmacist-led intervention groups relative to TAU: prescribing £112 (£24 to £200) and review £88 (−8 to £185). Controlling only for baseline costs and baseline SF-6D (ie, excluding age, sex, marital status, work status, education, income, baseline CPG—intensity) in regression analyses increased incremental costs for both pharmacist-led interventions relative to TAU: prescribing £125 (£82 to £242) and review £76 (−£40 to £192); this, however, had little impact on QALY gains relative to TAU: prescribing 0.0017 (−0.0127 to 0.0160) and review 0.0040 (−0.0099 to 0.0179)

### Expected value of sample information analysis

**Figure 2:**
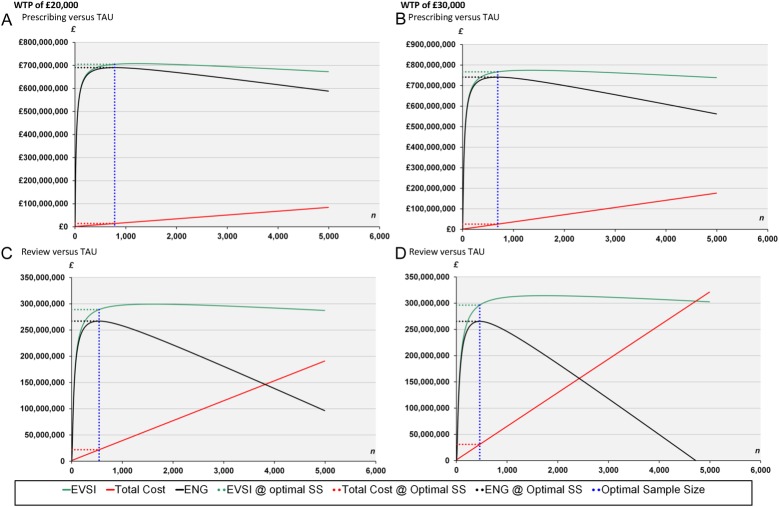
Value of expected value of sample information (EVSI) analysis. The expected net benefit of sampling at specified randomised controlled trial (RCT) sizes using a threshold of £20 000 per quality-adjusted life years (QALY) gained for (A) Prescribing versus treatment as usual (TAU), (C) Review versus TAU and using a threshold of £30 000 per QALY gained for (B) Prescribing versus TAU and (D) Review versus TAU. From regression analysis with adjustment for baseline costs, baseline short form six-dimension (SF-6D), and other patient characteristics (including age, sex, material status, education, work status, income chronic pain grade (CPG)-intensity). The number of participants with data on all these baseline variables: prescribing (n=35), review (n=39), TAU (n=34). ENG, expected net gain; EVSI, expected value of sample information; n, number of patients in each arm; SS, sample size.

The EVSI investigated the cost-effectiveness of carrying out a larger RCT of pharmacist prescribing and pharmacist review versus TAU. The parameters used in the EVSI calculation and the resulting expected EVSI is given in figure 2, which also shows the expected costs of the RCT and the resultant ENG. The expected costs of running the proposed trial rise linearly as the sample size (n) is increased; however, the expected benefits diminish as the sample size increases after a certain point, giving the optimal number of patients in each arm of the RCT. The ‘optimal trial size’ (SS) is estimated as 780 per arm (prescribing vs TAU comparison) and 540 per arm (review vs TAU comparison) using a cost per QALY threshold of £20 000 and 690 per arm (prescribing vs TAU comparison), and 460 per arm (review vs TAU comparison) using a cost per QALY threshold of £30 000.

The results of the sensitivity analyses around the main EVSI results (see table 4) showed that this finding was generally robust to assumptions about annual incidence and recruitment cost, but sensitive to annual accrual and the time horizon used.

## Discussion

This current study estimated the differences in mean costs and mean effectiveness (in terms of QALYs) of pharmacist medication review with or without prescribing as compared with usual GP care for the treatment of chronic pain in primary care based on data from the PIPPC pilot trial.^9^ We also assessed the EVSI for a definitive trial. The results suggested that the pharmacist interventions were likely to be more costly than TAU when differences in costs at baseline were taken into account, but there was a large degree of uncertainty surrounding the estimates of differences in mean costs and mean effects (ie, QALYs). Given this uncertainty, the economic case for pharmacist prescribing with or without review over TAU can only be established with a larger RCT. The potential gain from a future trial was estimated by the EVSI; this showed that the gains in monetary terms from a future trial exceed the expected costs.

In the main clinical paper,^9^ the CPG was shown to have potential to be able to discriminate between patients who improved postintervention and suggested maximum benefit for those in the pharmacist prescribing arm (effect size of 0.45).^9^ However, the difference in effect observed with the CPG was not reflected in a (large) effect difference in terms of QALYS. We believe a larger trial is now warranted to determine more precisely the true effects of pharmacist medication review with or without prescribing, measured in terms of CPG and QALYs.

**Table 4:** EVSI sensitivity analyses*

	WTP of £20 000 per QALY gained		WTP of £30 000 per QALY gained	
	Prescribing vs TAU	Review vs TAU	Prescribing vs TAU	Review vs TAU
Base case	780	540	690	460
Annual incidence halved	780	540	690	460
Annual incidence doubled	780	540	690	460
Recruitment cost halved	780	540	690	460
Recruitment cost doubled	780	540	690	460
Annual accrual rate halved	550	370	320	320
Annual accrual rate doubled	1110	770	670	670
Time horizon 10 years	300	300	250	250
Time horizon 20 years	440	430	370	370

*Estimates rounded to the nearest 10.

EVSI, expected value of sample information; TAU, treatment as usual; WTP, willingness to pay.

To the best of our knowledge, no other published studies have assessed the costs and effects (in terms of QALYs) of pharmacist interventions for chronic pain or pharmacist independent prescribing for any condition. A small feasibility study^19^ examining the impact of introduction of a combined nurse/pharmacist-led clinic for managing chronic pain in primary care was reported to lead to improvements in management of pain, as well as a reduction in use of secondary care resources and high rates of satisfaction. Stewart *et al*^20^ have argued for more research into the clinical, economic and humanistic effects of different forms of ‘non-medical prescribing’ (NMP). NMP involves the prescribing of medicines by healthcare professionals (who are not doctors, eg, nurses, pharmacists) but who have undergone appropriate training (including pharmacovigilance) and are, therefore, qualified to prescribe either in a supplementary or independent role within their areas of competence.^20^ Our study results provide new information about the costs and QALY effects of pharmacist medication review with or without prescribing for chronic pain.

One important aspect of the design of the cost effectiveness component of RCTs—including those evaluating pharmacist interventions for pain—relates to the selection of the most appropriate health utility measures. The SF-6D was used in the current trial. An alternative is the EQ-5D and this has become the instrument of choice for many agencies including the National Institute for Health and Care (formerly ‘Clinical’) Excellence (NICE).^21^ The SF-6D cannot easily be compared with the EQ-5D because of differences in their descriptive systems, values applied to health states and contextual basis (ie, recall period). Moreover, a number of studies have reported that these two measures cannot be used interchangeably in patients with chronic pain.^22–27^ In a study of 389 patients with chronic knee pain, Barton *et al*^28^ reported that, in contrast to the EQ-5D, the SF-6D was unable to discriminate between patients who improved postintervention and those who did not; but in another study of patients with chronic low back pain and degenerative disc disease,^25^ the SF-6D had the best ability to detect changes and correctly identify patients as improved or non-improved. In a UK general population study,^29^ both measures were shown to discriminate between those self-reporting chronic pain or no chronic pain with and without neuropathic characteristics. However, both measures generated widely different health utility scores for the same patient groups, for example, the mean utilities for severe pain (EQ-5D 0.33 vs SF-6D 0.58). In another study,^30^ the EQ-5D was found to be more responsive to deterioration in health, whereas the SF-6D was more responsive to improvement in health in patients with knee pain due to inflammatory arthritis. The choice of instrument might have a considerable impact on the conclusions reached about the cost-effectiveness of the interventions being evaluated. For instance, a Norwegian cost-effectiveness study of total disc replacement versus multidisciplinary rehabilitation in patients with chronic low back pain found that total disc replacement was cost-effective when EQ-5D was used, but not when SF-6D was used.^26^ Additionally, the practical issue of instrument completion is important in future trial design. In our analysis, we found around one-third of SF-6D measurements were incomplete. Similar results have been found in other studies in patients with pain, where rates of completion were significantly better for the EQ-5D over the course of an RCT in patients with non-specific neck pain^27^ and in patients with low back pain and degenerative disease.^25^ Finally, it should be noted that a new 5-level version of the EQ-5D has recently been launched^31^ and its application in patients with various chronic conditions (eg, osteoarthritis) suggests it might have improved discriminative capacity and sensitivity to change than the EQ-5D 3-level version.^32^ The use of both the EQ-5D and the SF-6D might be the best approach in future trials in patients with chronic pain.

In conclusion, the present study suggests that pharmacist-led medication review with or without prescribing in patients with chronic pain in primary care had similar effects in terms of QALYs compared with treatment as usual but was more expensive. These results, however, are highly uncertain due to the small sample size of the pilot trial and do not reflect the previously reported significantly improved chronic pain grade. The EVSI analysis indicated that this current evidence is insufficient for decision-making and that a future larger trial is worthwhile. Such a trial would require between 460 and 690 patients and between 540 and 780 patients per arm using a threshold of £30 000 and £20 000 per QALY gained, respectively, in order to obtain more precise estimates of differences in mean effects (QALYs) and mean costs between treatment groups.
